# Community Pharmacists' Knowledge and Perception towards Telepharmacy Services and Willingness to Practice It in Light of COVID-19

**DOI:** 10.1155/2024/6656097

**Published:** 2024-01-31

**Authors:** Ayesha Siddiqua, Soha Makki, Sazada Siddiqui, Randa A. Abdelkarim, Tahani Jubran, Wejdan Nwar, Ahlam Alqahtani, Maryam Alshehri, Maram Saeed, Arwa Khaled

**Affiliations:** ^1^Department of Clinical Pharmacy, College of Pharmacy, King Khalid University, Abha, Saudi Arabia; ^2^Department of Biology, College of Science, King Khalid University, Abha, Saudi Arabia; ^3^Faculty of Mathematical Sciences and Informatics, University of Khartoum, Khartoum, Sudan; ^4^Department of Clinical Pharmacy, Beni-Suef University Hospital, Beni-Suef University, Beni Suef, Egypt

## Abstract

**Background:**

During the COVID-19 pandemic, there was increased adoption of telepharmacy, which has proven benefits. This study was conducted to assess the knowledge and perception of the community pharmacists of Aseer region, Saudi Arabia, towards telepharmacy services and evaluate their willingness to adopt telepharmacy in clinical practice during the COVID-19 pandemic.

**Materials and Methods:**

A cross-sectional study was conducted using an online self-administered structured anonymous questionnaire on the community pharmacists of Aseer region, Saudi Arabia. It covers demographics, computer access and literacy, knowledge and perception, and willingness to practice telepharmacy. Results were expressed as frequencies, percentages, and mean. The comparison between the classes of the demographic variables and the scores was done via Kruskal–Wallis and Mann–Whitney tests.

**Result:**

About half of the pharmacists in our study showed average knowledge about telepharmacy, the practical application of telepharmacy technology, and telepharmacy guidelines with a percentage of 53%, 52%, and 47%, respectively). Majority of the participants showed high perception towards telepharmacy in Saudi Arabia, while 93% of the pharmacists in Saudi Arabia agreed that the implementation of telepharmacy technology is appropriate due to the current COVID-19 pandemic. Only a significant relation was found between gender and computer access, literacy, and perceptions towards telepharmacy with *p* values of 0.033 and 0.026, respectively.

**Conclusion:**

The majority of the community pharmacists exhibited a positive perception and are willing to practice the concept of telepharmacy despite having a below average knowledge of telepharmacy. A future study involving the entire kingdom of Saudi Arabia could help identify the gaps in the knowledge, perception, and willingness to practice telepharmacy on a broader scale and thus promote telepharmacy adoption in the entire kingdom.

## 1. Introduction

Before the health crisis, virtual care services underwent a gradual upsurge to provide pharmaceutical care services to patients in remote areas with limited healthcare accessibility and facilities. However, due to the coronavirus disease 2019 (COVID-19) pandemic, there was increased adoption of telehealth, where patients communicate with the healthcare providers remotely for their healthcare needs and concerns by using technology [1–3]. One area that comes under the umbrella of telehealth is telepharmacy, in which pharmacists provide patient care activities that improve the patients' safety and quality of life by reducing medication errors, adverse drug events, and reduced healthcare costs [2, 4, 5]. Community pharmacists, who are a vital part of the healthcare system and are easily accessible, could play a prominent role in providing benefits to the community and improving the patients' health outcomes during the COVID-19 pandemic through this new concept of telepharmacy. The National Association of Boards of Pharmacy described telepharmacy as “the provision of pharmaceutical care to patients at a distance through the use of telecommunication and information technologies” [6, 7]. In the past few years, telepharmacy has been practiced in various hospitals with proven benefits like enhancing patients' adherence towards medication use by providing sufficient medication counseling time with privacy, thus increasing patients' trust and satisfaction. For example, in a recent study conducted on remote hospital inpatients in Australia, Queensland, the concept of telepharmacy helped in providing medication-related advice and counseling [8]. A study conducted on Canadian pharmacists reported that telepharmacy improved Canadian pharmacists' clinical practice, and they felt comfortable providing suggestions for mild ailments. Users' main reasons for using telepharmacy were that it was easier to set up the system, that privacy and data protection were better, and that the technology was easy to learn [9]. Another study conducted in Spain on pharmacist teleconsultation of 38 patients reported a high level of patient satisfaction and achievement of treatment goals [10]. A similar cross-sectional study conducted in Nigeria reported good acceptability of the concept by patients who were also willing to pay a moderately acceptable amount for the services [11]. Along with proven benefits, several barriers have also been reported in the healthcare setting in the past. Some of them were lack of knowledge and training of employees, difficulty in adopting new technology, expenses to implement, and patients' acceptance, which may affect the practice of telepharmacy. Pharmacists' knowledge and perception of telepharmacy determines the success and willingness to adopt telepharmacy in the pharmacy field [12]. So, it is essential to explore users' views about this novel technology. A recent study conducted in Riyadh city of Saudi Arabia assessed the knowledge and attitude of pharmacists towards telepharmacy; this study reported that the pharmacists exhibited poor knowledge but showed a positive attitude towards telepharmacy services and with adequate education and training, telepharmacy could be incorporated by the pharmacists into the healthcare system of Saudi Arabia [4]. Another study conducted in Jordan reported that the community and hospital pharmacists showed a favorable attitude towards practicing this novel technology and emphasized the need to practice this telepharmacy technology as this COVID-19 may take time to resolve completely [13]. Considering the pandemic situation where social distancing had become a norm and the rise in need for telepharmacy services to improve the quality of pharmaceutical services, this study aimed to assess the knowledge and perception of the community pharmacists of Aseer region, Saudi Arabia, towards telepharmacy services and evaluate their willingness to adopt telepharmacy in clinical practice during the COVID-19 pandemic.

## 2. Materials and Methods

### 2.1. Study Design and Setting

A three-month cross-sectional study was conducted using an online self-administered structured anonymous questionnaire on the community pharmacists of Aseer region, Saudi Arabia.

### 2.2. Study Population, Sample Size, and Sampling Criteria

This study targeted the community pharmacists of Aseer region, Saudi Arabia. There are a total of 747 licensed community pharmacists across 438 licensed community pharmacies [14]. The estimated minimum recommended sample size was 254 calculated by Raosoft, sample size calculator, with 95% confidence interval and 5% precision limit. The inclusion criteria were practicing licensed Saudi and non-Saudi community pharmacists of both genders of Aseer region, Saudi Arabia. The exclusion criteria were pharmacists from other sectors such as academics, hospitals, industries, pharmaceutical companies, and those not willing to participate. This study used snowball sampling to recruit eligible participants. Initially, we sent the survey link to some community pharmacists, and they forwarded the survey link to the other pharmacists.

### 2.3. Survey Instrument and Data Collection

An online self-administered structured anonymous questionnaire based on instruments used in a previous study of Albarrak et al. [7] was adapted by conducting an extensive literature review. Focus group discussions were held by conducting an informal online discussion with participants to understand their perceptions, beliefs, and information needs regarding this new technology service. Suggestions were taken, and a draft questionnaire was prepared to fulfill the objective of this study. It was then subjected to face and content validation where an informal review of a questionnaire by nonexperts was used to assess its clarity, comprehensibility, and appropriateness for the target group, as well as a formal assessment by subject experts to determine the appropriateness of the content and identify any misunderstandings or omissions. The questionnaire was translated into Arabic for the participants' convenience and to avoid ambiguity. Even though the target population was community pharmacists, who are typically able to understand English, the questionnaire was translated into Arabic for the convenience of the participants and to eliminate any confusion they might encounter, as this was a self-administered questionnaire in which they will read, understand, and respond to the questions on their own. The accuracy in translation was checked by researchers who were proficient in the native language and a pilot study was conducted. The questionnaire was piloted with ten respondents representing the study population to determine the clarity of the language and the structure of the questionnaire. The pilot study results were not included in the results; nevertheless, the feedback was analyzed, the double-barreled, confusing, and leading questions were modified, and a finalized questionnaire was created.

The questionnaire consists of five sections comprising 4 questions on demographic characteristics and general information such as age, gender, nationality, and work experience, 6 questions on general information about access to computers and their literacy, 5 questions each on knowledge and perception, and 3 questions assessing the willingness to practice telepharmacy. The internal consistency of the knowledge, perception, and willingness to practice sections were assessed with Cronbach's alpha value each of 0.834, 0.792, and 0.677, respectively, indicating that the questions were reliable. The scoring for the section on general information about access to computers and their literacy was measured as always or often = 2, sometimes or rarely = 1, and never = 0. A 3-point Likert scale was used to assess the pharmacist's knowledge (low = 0, average = 1, and high = 2), whereas a 2-point scale was used to measure perception and willingness; agree = 1 and disagree = 0. The overall knowledge, perception, and willingness to practice were measured by the original Bloom's cutoff points, 80.0–100.0%, 60.0–79.0%, and ≤59.0%, which were adapted and modified from the 2020 study by Khaled et al. [15].

The participants' total knowledge score (maximum: 10) was classified as above average (8–10), average (6–7.9), or below average (<6). The perception section's total score (maximum: 5) was classified as positive (4-5 score), moderate (3–3.95 score), or negative (<2.95). For the willingness to the practice section, the total score (maximum: 3) was classified as willing for 2.4–3, moderately willing for 1.8–2.3, and not willing for <1.7.

### 2.4. Statistical Analysis

After data collection, responses were downloaded in MS Excel file and assessed blindly. Results were expressed as frequencies, percentages, and mean (descriptive statistics). The comparison between the classes of the demographic variables and the scores was done via nonparametric tests named Kruskal–Wallis (age and work experience) and Mann-Whitney (gender) (SPSS Inc, Chicago, IL, USA). A *p* value <0.05 was considered significant.

### 2.5. Ethical Considerations

The Research Ethics Committee of King Khalid University approved this project, with approval number ECM# 2021-5627. The consent of participants was requested and recorded on the very first page of the survey, demonstrating their voluntary participation.

## 3. Results

The study received 241 responses (response rate: 94.8%) of which some responses were incomplete, so excluding them from the analysis, the new total response was 228. The demographics of the participants are presented in Table 1. Majority of the participants were female and most of them were reported to have less than 5 years of work experience as community pharmacists and were from the age group of 21–40 years.

### 3.1. Pharmacists' Computer Access and Literacy

To adopt telepharmacy into practice, computer access and literacy are a necessity.  Table 2 displays the responses related to the pharmacists' computer access and literacy. 63% of the participants always use PC/laptop at home. Meanwhile, 32% often interact with patients via e-mail or through social media, 43% of them always use social media Internet for obtaining information to give to patients, only 39% use social media Internet for patient consultation, and 71% use social media Internet to update their knowledge and skills.

### 3.2. Pharmacists' Knowledge on Telepharmacy

About half of the pharmacists in our study showed average knowledge about telepharmacy, the practical application of telepharmacy technology, and telepharmacy guidelines with a percentage of 53%, 52%, and 47%, respectively. Meanwhile, 45% showed that average response towards the impact of the continuous training in the use of telepharmacy is necessary for pharmacists and 43% had conferences, speeches, or meetings held in their workplace regarding telepharmacy technology. The responses of pharmacists regarding their knowledge of telepharmacy are presented in Table 3.

### 3.3. Pharmacists Perception of Telepharmacy

Majority of the participants showed high perception towards telepharmacy in Saudi Arabia. About 94% agreed that use of telepharmacy system during the COVID-19 pandemic can help patients. 91% of the pharmacists believed that there is a potential role for ICT in the healthcare. 89% of the participants agreed that telepharmacy is a viable approach for providing pharmaceutical care services to patients. 86% of the participants believed in the time-saving effect of telepharmacy (Table 4).

### 3.4. Willingness to Practice Telepharmacy

93% of the pharmacists in Saudi Arabia agreed that the implementation of telepharmacy technology is appropriate due to the COVID-pandemic. 91% of the participants would like to consult with the large centers in their specialty, whilst they are in their own pharmacies. 86% of the respondents agreed that the telepharmacy system can be integrated within the existing system (Table 5).

### 3.5. Regarding the Issues Affecting the Adoption of Telepharmacy

The participants were asked about the issues that may affect the adoption of telepharmacy, and their responses are listed in Table 6.

86 community pharmacists showed concern about patient privacy and confidentiality, and 30% were worried about the availability of user-friendly software. Also, 27% showed concerns about the lack of consultation between information technology experts and clinicians, while 25% of them were worried about the perceived increase in workload.

Association between the demographic factors of the respondents and the computer literacy, knowledge, perception, and willingness using the Kruskal-Wallis/Mann-Whitney test showed that no significant association was noted between the participants with different age, different nationality, or different years of experience; there is only a significant relation between gender and computer access, literacy, and perceptions towards telepharmacy with *p* values of 0.033 and 0.026, respectively (Table 7).

Table 8 reflects the mean score results of computer access and literacy, knowledge, attitude, and willingness to practice telepharmacy. Using Blooms cutoff points, it was obvious that the participants displayed a positive perception towards the adoption of telepharmacy, and they were willing to practice telepharmacy despite having a below-average score of knowledge.

## 4. Discussion

The impact of the COVID-19 outbreak on direct access to pharmaceutical services has boosted global interest in telepharmacy. The purpose of this study was to investigate community pharmacists' knowledge, perception, and willingness to practice telepharmacy. According to the findings, the study participants had below-average knowledge, which differs from the study in Riyadh by Alanazi et al. [16]. The introduction of COVID-19, according to Unni et al., has accelerated the necessary modifications to make telepharmacy an appealing option. They also claimed that as healthcare practitioners and the general public become more aware of the benefits of telepharmacy, it may persist long after the pandemic [17]. According to Ibrahim et al., pharmacies that use these tele services may be able to serve COVID-19 patients more quickly than pharmacies that do not use them [18]. In response to the COVID-19 pandemic in a city of Vietnam, Dat et al. reported that 87% of the pharmacists used telepharmacy in their pharmacy practice. They also claimed that giving medical information and remote medicine guidance had become a compulsion in the context of the COVID-19 outbreak [19]. Even though the pharmacists might not have enough knowledge about telepharmacy now, but due to this need to practice and continuous exposure, they might be forced to increase their knowledge on telepharmacy.

Whereas regarding their perception, the current study discovered that community pharmacists had a favorable perception towards telepharmacy adoption, which is similar to a study conducted on Jordanian pharmacists by Muflih et al., 2021 [13]. According to 89% of the participants, telepharmacy is a realistic strategy for providing pharmaceutical care services to patients. Telepharmacy allows patients to avoid waiting at a clinic with other sick patients, save time traveling, avoid work loss, and get themselves and their family healthy when possible. According to 86% of the pharmacists in the current study, telepharmacy saves patients money and time by eliminating the need for them to visit healthcare facilities. Previous research has shown that telepharmacy reduces travel costs and saves time, which is a significant barrier for patients receiving healthcare in rural and remote settings, particularly the disabled and elderly [20]. In Alanazi et al.'s study, more than 91% of the pharmacists believed that using a telepharmacy system would save them time and money [16]. Telepharmacy and telehealth clinical pharmacy services help bridge the gap in rural locations where pharmacists and physicians are in short supply. In contrast to the study conducted by Liu et al. in China, this study indicated that the study participants were willing to provide remote services to their patients [21]. Participants in a study done by Ahmed et al. were shown to be less prepared for telepharmacy [22]. According to Elnaem et al., around 67% of the senior pharmacy students at a Malaysian public pharmacy school displayed excellent knowledge and 68% demonstrated high readiness. They also discovered that characteristics, including a lack of enthusiasm and an extreme workload, were related to participants' telepharmacy preparedness [23]. Payment and reimbursement concerns, as well as a lack of access to information technology infrastructure, were among the most significant hurdles, according to Ameri et al. [24]. Dat et al. found that roughly 87.2% of the pharmacists in their survey were willing to employ telepharmacy [19]. Pharmacy schools should incorporate telepharmacy practice models into their curricula to better train future pharmacists to provide telepharmacy services. Furthermore, pharmacists' expertise and preparedness to use telepharmacy can be strengthened through presenting lectures, workshops, and attending conferences. Continuing professional education and intense training may aid in increasing knowledge, which may improve community pharmacists' perception and willingness to practice. The availability of appropriate and efficient resources, as well as the supply of incentives to pharmacists, would aid in the promotion of telepharmacy implementation [22].

### 4.1. Study Limitations

This study has included participants from only the Aseer region, so the results cannot be generalized to all the community pharmacists of Saudi Arabia, but this can be overcome by expanding this study to the entire kingdom in the future. The participants were recruited by snowball sampling to reach the community pharmacists working in the remote areas of the Aseer region; however, there might be a possibility of sampling bias.

## 5. Conclusion

This study concludes that the majority of the community pharmacists exhibited a positive perception and are willing to practice the concept of telepharmacy despite having a below average knowledge of telepharmacy. As telepharmacy holds significant promise and community pharmacists are the most frequently and easily approachable healthcare professionals, they should be encouraged to implement such technologies and be well trained by identifying the gaps. A future study involving the community pharmacists of the entire kingdom of Saudi Arabia could help identify the gaps in the knowledge, perception, and willingness to practice telepharmacy on a broader scale and thus promote telepharmacy adoption in the entire kingdom as there is an immense need for such remote service due to its convenience in providing remote healthcare services to patients, especially during the COVID-19 pandemic or any other epidemics that may arise in the future.

## Figures and Tables

**Table 1:** Demographic detail.

		*N* (228)	%
Age	21–30	111	49
	31–40	72	32
	41–50	39	17
	>50	6	3

Gender	Female	125	55
	Male	103	45

Nationality	Non-Saudi	61	27
	Saudi	167	73

Work experience as a community pharmacist in (years)	<5 years	136	60
	5–15 years	70	31
	>15 years	22	10

*N* = No. of participants', % = percentage of participants.

**Table 2:** Computer access and literacy.

		*N* (228)	%
How often do you use PC/laptop at home?	Always or often	144	63
	Sometimes or rarely	78	34
	Never	6	3

As a pharmacist, how often do you interact with patients via e-mail or through social media?	Always or often	72	32
	Sometimes or rarely	93	41
	Never	63	28

Have you been questioned by patients about online means of contacting you?	Always or often	60	26
	Sometimes or rarely	99	43
	Never	69	30

Do you use social media Internet for obtaining information to give to patients?	Always or often	99	43
	Sometimes or rarely	101	44
	Never	28	12

Do you use social media/Internet for patient consultation?	Always or often	90	39
	Sometimes or rarely	93	41
	Never	45	20

Do you use social media Internet to update your knowledge and skills?	Always or often	162	71
	Sometimes or rarely	53	23
	Never	13	6

*N* = No. of participants', % = percentage of participants.

**Table 3:** Knowledge of telepharmacy.

		*N* (228)	%
To what extent are you familiar with telepharmacy technology?	High	45	20
	Average	121	53
	Low	62	27

To what extent are you familiar with the practical application of telepharmacy technology?	High	44	19
	Average	119	52
	Low	65	29

How often have conferences, speeches, or meetings held in your workplace regarding telepharmacy technology?	High	34	15
	Average	97	43
	Low	97	43

To what extent are you familiar with telepharmacy guidelines?	High	43	19
	Average	108	47
	Low	77	34

To what extent is continuous training in the use of telepharmacy necessary for pharmacists?	High	88	39
	Average	103	45
	Low	37	16

*N* = No. of participants', % = percentage of participants.

**Table 4:** Perception towards telepharmacy.

		*N* (228)	%
Telepharmacy is a viable approach for providing pharmaceutical care services to patients	Agree	203	89
	Disagree	25	11

There is a potential role for ICT (information and communications technology) in the healthcare	Agree	207	91
	Disagree	21	9

Using of the telepharmacy system can save time and money	Agree	192	84
	Disagree	36	16

Using of the telepharmacy system during the COVID-19 pandemic can help patients	Agree	215	94
	Disagree	13	6

Telepharmacy system can save efforts	Agree	196	86
	Disagree	32	14

**Table 5:** Willingness to practice telepharmacy.

		*N* (228)	%
I would like to consult with the large centers in my specialty, whilst I am in my own pharmacy	Agree	207	91
	Disagree	21	9

The implementation of telepharmacy technology is appropriate due to the COVID pandemic	Agree	211	93
	Disagree	17	7

Telepharmacy system can be integrated within the existing system	Agree	195	86
	Disagree	33	14

**Table 6:** Issues affecting the adoption of telepharmacy.

		*N* (228)	%
What are the issues affecting the adoption of telepharmacy	Concern about patient privacy and confidentiality	86	38
	Lack of user-friendly software	69	30
	Lack of consultation between information technology experts and clinicians	61	27
	Negative attitudes of staff involved	66	29
	Perceived increase in workload	56	25

**Table 7:** Association between demographic characteristics and the computer access and literacy, knowledge, perception, and willingness to practice telepharmacy.

	Computer access and literacy	Knowledge of telepharmacy	Perception towards telepharmacy	Willingness to practice telepharmacy
Age	0.063	0.210	0.069	0.061
Gender	0.033^*∗*^	0.333	0.026^*∗*^	0.099
Work experience as a community pharmacist in (years)	0.150	0.075	0.184	0.310

*p* value <0.05 is significant.

**Table 8:** Mean score results.

	Mean	Standard deviation	Maximum	Minimum	Mode (most achieved)
Knowledge of telepharmacy	4.6	2.86	10.00	0.00	5
Perception towards telepharmacy	4.4	1.16	5.00	0.00	5
Willingness to practice telepharmacy	2.7	0.71	3.00	0.00	3

## Data Availability

The datasets used to support the findings of this study are not publically available due to privacy concerns but are available upon reasonable request from the corresponding author.

## References

[1] Hasani S., Ghafri T., Al Lawati H. (2020). The use of telephone consultation in primary health care during COVID-19 pandemic, Oman: perceptions from physicians. *Journal of Primary Care and Community Health*.

[2] Manuel F. C., Wieruszewski E. D., Brown C. S., Russi C. S., Mattson A. E. (2022). Description of telepharmacy services by emergency medicine pharmacists. *American Journal of Health-System Pharmacy*.

[3] Iftinan G. N., Elamin K. M., Rahayu S. A., Lestari K., Wathoni N. (2023). Application, benefits, and limitations of telepharmacy for patients with diabetes in the outpatient setting. *Journal of Multidisciplinary Healthcare*.

[4] Alfadl A., Alanazi A., Hussain A. (2016). Pharmaceutical care in the community pharmacies of Saudi Arabia: present status and possibilities for improvement. *Saudi Journal of Medicine and Medical Sciences*.

[5] Mohamed Ibrahim O., Ibrahim R. M., Abdel-Qader D. H., Al Meslamani A. Z., Al Mazrouei N. (2021). Evaluation of telepharmacy services in light of COVID-19. *Telemedicine and E-Health*.

[6] Bonner L. (2020). Telehealth basics for pharmacists during COVID-19 and beyond. *Pharmacy Today*.

[7] Albarrak A., Mohammed R., Almarshoud N. (2021). Assessment of physician’s knowledge, perception and willingness of telemedicine in Riyadh region, Saudi Arabia. *Journal of Infection and Public Health*.

[8] McFarland R. (2017). Telepharmacy for remote hospital inpatients in north-west Queensland. *Journal of Telemedicine and Telecare*.

[9] Park J. Y., Zed P. J., A De Vera M. (2022). Perspectives and experiences with telepharmacy among pharmacists in Canada: a cross-sectional survey. *Pharmacy Practice*.

[10] Margusino-Framiñán L., Cid-Silva P., Castro-Iglesias Á (2019). Teleconsultation for the pharmaceutical care of HIV outpatients in receipt of home antiretrovirals delivery: clinical, economic, and patient-perceived quality analysis. *Telemedicine and e-Health*.

[11] Nduka S. O., Nwaodu M. A., Nduka I. J. (2022). Telepharmacy services in a developing country: nigerian community pharmacists’ and patients’ perspectives on the clinical benefits, cost, and challenges. *Telemedicine and E-Health*.

[12] Biruk K., Abetu E. (2018). Knowledge and attitude of health professionals toward telemedicine in resource-limited settings: a cross-sectional study in north west Ethiopia. *Journal of Healthcare Engineering*.

[13] Muflih S., Al‐Azzam S., Abuhammad S., Jaradat S., Karasneh R., Shawaqfeh M. (2021). Pharmacists’ experience, competence and perception of telepharmacy technology in response to COVID‐19. *International Journal of Clinical Practice*.

[14] Alsayari A., Almghaslah D., Khaled A. (2018). Community pharmacists’ knowledge, attitudes, and practice of herbal medicines in asir region, kingdom of Saudi Arabia. *Evidence-based Complementary and Alternative Medicine*.

[15] Khaled A., Siddiqua A., Makki S. (2020). The knowledge and attitude of the community from the aseer region, Saudi Arabia, toward COVID-19 and their precautionary measures against the disease. *Risk Management and Healthcare Policy*.

[16] Alanazi A., Albarrak A., Alanazi A., Muawad R. (2021). 5PSQ-184 Knowledge and attitude assessment of pharmacists toward telepharmacy in Riyadh, Saudi Arabia. *European Journal of Hospital Pharmacy*.

[17] Unni E. J., Patel K., Beazer I. R., Hung M. (2021). Telepharmacy during COVID-19: a scoping review. *Pharmacy*.

[18] Ibrahim O. M., Ibrahim R. M., Z Al Meslamani A., Al Mazrouei N. (2023). Role of telepharmacy in pharmacist counselling to coronavirus disease 2019 patients and medication dispensing errors. *Journal of Telemedicine and Telecare*.

[19] Dat T. V., Tran T. D., My N. T. (2022). Pharmacists’ perspectives on the use of telepharmacy in response to COVID-19 pandemic in Ho chi minh city, Vietnam. *Journal of Pharmacy Technology*.

[20] Telepharmacy W. A. Z. (2017). Time to pick up the line. *Research in Social and Administrative Pharmacy*.

[21] Liu S. L. P., Luo P., Tang M. (2020). Providing pharmacy services during the coronavirus pandemic. *International Journal of Clinical Pharmacy*.

[22] Ahmed N. J., Almalki Z. S., Alsawadi A. H. (2023). Knowledge, perceptions, and readiness of telepharmacy among hospital pharmacists in Saudi Arabia. *Healthcare*.

[23] Elnaem M. H., Akkawi M. E., Al-Shami A. K., Elkalmi R. (2022). Telepharmacy knowledge, perceptions, and readiness among future Malaysian pharmacists amid the COVID-19 pandemic. *Indian Journal of Pharmaceutical Education and Research*.

[24] Ameri A., Salmanizadeh F., Keshvardoost S., Bahaadinbeigy K. (2020). Investigating pharmacists’ views on telepharmacy: prioritizing key relationships, barriers, and benefits. *Journal of Pharmacy Technology*.

